# Association between FTO gene polymorphisms and HDL cholesterol concentration may cause higher risk of cardiovascular disease in patients with acromegaly

**DOI:** 10.1007/s11102-017-0840-8

**Published:** 2017-09-14

**Authors:** Aleksandra Franczak, Katarzyna Kolačkov, Aleksandra Jawiarczyk-Przybyłowska, Marek Bolanowski

**Affiliations:** Department of Endocrinology, Diabetes and Isotope Therapy, Wroclaw Medical University, Pasteura 4, 50-367 Wroclaw, Poland

**Keywords:** Acromegaly, FTO gene, Cardiovascular disease, Rs9939609, Rs9930506

## Abstract

**Introduction:**

Cardiovascular diseases are main cause of morbidity and mortality in acromegaly. Polymorphisms of *FTO* gene are associated with obesity and increased risk of CVD (independently of BMI). Aim of this study was to investigate the allele frequencies of two *FTO* gene polymorphisms: rs9939609 and rs9930506 in patients with acromegaly and to examine the association of *FTO* gene polymorphisms with BMI and selected metabolic parameters.

**Materials and methods:**

Identification of two single nucleotide polymorphisms of FTO gene was carried out in 51 patients with acromegaly using the minisequencing method.

**Results:**

The risk-allele frequencies of rs9939609 and rs9930506 polymorphisms were 0.471 and 0.529, respectively and they were higher than in general European population. There is no association of *FTO* gene polymorphisms with BMI, glucose, total cholesterol, LDL cholesterol and triglyceride. The risk alleles were associated with decreased HDL cholesterol concentration. Homozygotes for the rs9939609-risk allele had 1.25-fold lower HDL cholesterol concentration than carriers of the TT genotype (p = 0.0024). The estimated average decrease in HDL cholesterol concentration per risk allele for rs9930506 was 11.2%. Nevertheless, statistically significant differences were observed only between AG versus GG and AA versus GG genotypes. Homozygotes for the rs9930506-risk allele had 1.27-fold lower HDL cholesterol concentration than carriers of the AA genotype (p = 0.007).

**Conclusion:**

The risk-allele frequencies of studied polymorphisms in acromegaly were higher than in general European population. There is an association between *FTO* gene polymorphisms and HDL cholesterol concentration, suggesting *FTO* gene polymorphisms may be associated with higher CVD risk in patients with acromegaly.

## Introduction

Acromegaly is a chronic disease caused by excessive growth hormone (GH) secretion from pituitary adenoma and secondary elevation of insulin-like growth factor 1 (IGF-1) concentration. Despite of characteristic features, the diagnosis of acromegaly is usually delayed by 5–10 years after the first symptoms. As a consequence of prolonged GH and IGF-1 actions various complications causing deteriorated quality of life and premature mortality are observed. Among them are cardiovascular and respiratory diseases, neoplasm, arthropathy and metabolic disorders [1, 2]. The leading causes of death in patients with acromegaly are cardiovascular diseases (CVD) including heart valve disease, hypertension, cardiac arrhythmia and coronary artery disease. Moreover, acromegalics have an increased prevalence of some cardiovascular risk factors such as dyslipidemia, hypertension and impaired glucose tolerance or diabetes. The main risk factors for cardiac death among patients with acromegaly are age, metabolic disorders and prolonged increased GH and IGF-1 concentrations [3].

The advancement of molecular biology techniques allowed for the examination of the genetic background of many diseases. Thanks to Genome-Wide Association Studies (GWAS), lots of genes and their polymorphisms were identified as associated with some diseases or measurable traits [4]. In 2007, two research groups independently identified common genetic variants in fat mass and obesity related (*FTO*) gene which predisposes to diabetes through an effect on obesity-related traits. According to Frayling et al. [5] homozygotes for the risk allele had 1.67-fold increased risk of obesity and weighted about 3 kg more when compared with those who did not inherit a risk allele. Scuteri et al. [6] indicated that in Sardinian population homozygotes for risk allele were 1.3 body mass index (BMI) units heavier than homozygotes for another allelic variant. Further studies suggested the association of polymorphisms in the *FTO* gene with CVD risk, independently of BMI and other conventional CVD risk factors [7–9]. *FTO* gene is a polymorphic gene located on chromosome 16 at position 16q12.2. This gene encodes a 2-oxoglutarate-dependent nucleic acid demethylase which is involved in DNA repair and fatty acid metabolism. The Fto protein catalyses the demethylation of single-stranded nucleic acids. The *FTO* mRNA is abundant in many tissues, particularly in the hypothalamic nuclei controlling energy balance. Some researches indicate that the *FTO* gene plays a role in nervous and cardiovascular systems although its biological function is not fully known yet. It is suggested that catalytic activity of *FTO* may regulate the transcription of genes involved in fatty acid and glucose metabolism [10, 11].

Furthermore, it is suggested that IGF-1, which is the main mediator of growth hormone actions, may also mediate actions of *FTO* gene [12]. So far there is no data concerning the influence of *FTO* gene polymorphisms on cardiovascular system and associated metabolic disturbances in acromegalic patients.

The aim of this study was therefore to investigate the risk allele frequencies of two *FTO* gene polymorphisms: rs9939609 and rs9930506 in patients with acromegaly and to examine the association of *FTO* gene polymorphisms with BMI and selected metabolic parameters.

## Materials and methods

### Study group

A total of 51 patients with acromegaly (37 female and 14 male), hospitalized in Clinic of Endocrinology, Diabetes and Isotope Therapy, Wroclaw Medical University, were included in the study. Written informed consent for participation in the study was obtained. The group underwent physical examinations, anthropometric measurements and provided a blood sample for biochemistry and genetic analysis. The project was approved by the Bioethics Committee of Wroclaw Medical University (no. KB-291/2015). The general characteristics of the study group are presented in Table 1.

**Table 1:** Characteristics of patients with acromegaly

Parameters (Mean ± SD)	All patients (n = 51)	Women (n = 37)	Men (n = 14)
Age (years)	56.0 ± 15.1	57.4 ± 16.2	52.4 ± 11.6
Body mass (kg)	81.1 ± 13.1	77.9 ± 13.1	89.5 ± 8.88
Height (m)	1.70 ± 0.1	1.66 ± 0.06	1.79 ± 0.1
BMI (kg/m^2^)	28.2 ± 4.5	28.3 ± 4.9	28.0 ± 3.3
Glucose (mg/dl)	97.2 ± 20.5	98.0 ± 23.4	95.2 ± 9.4
Total cholesterol (mg/dl)	205.5 ± 44.7	202.2 ± 49.3	214.2 ± 29.0
Triglyceride (mg/dl)	118.0 ± 52.5	119.6 ± 56.8	113.5 ± 40.6
HDL-C (mg/dl)	54.7 ± 14.1	54.3 ± 15.2	55.6 ± 10.9
LDL-C (mg/dl)	127.2 ± 38.8	124.0 ± 41.7	135.9 ± 29.3

*BMI* body mass index, *HDL-C* high-density lipoprotein-cholesterol, *LDL-C* low-density lipoprotein-cholesterol

### Anthropometric and laboratory measurements

Height and body mass were used to calculate body mass index. Plasma glucose, serum triglyceride, HDL, LDL and total cholesterol were determined from the blood. Glucose concentration was assessed by enzymatic method with glucose oxidase (Dade Behring GmbH, Germany), Total cholesterol, HDL-cholesterol and triglyceride were analyzed using enzymatic method (SPINREACT, San Esteve de Bas, Spain). LDL-cholesterol concentration was calculated using Friedewald formula [13].

### Genotyping

The genomic DNA was extracted from peripheral blood leukocytes in the blood samples according to the manufacturer’s instructions (NucleoMag^®^ 96 Blood Isolation Kit, Macherey-Nagel, Germany). Specific fragments of the *FTO* gene were amplified by polymerase chain reaction (PCR) using the TaKaRa Taq DNA Polymerase Amplification Kit (Takara Bio Inc., Japan). Optimization of PCR conditions was conducted to amplify simultaneously both of specific gene fragments containing the polymorphic site. The reactions were carried out in the presence of specifically designed pair of primers (synthesized by Generi Biotech s.r.o., Czech Republic). The amplification was performed using a TPersonal Termocycler (Biometra GmbH, Germany). The identification of polymorphisms was conducted using the minisequencing method according to the protocol of an ABI PRISM^®^ SNaPshot™ Multiplex Kit (Thermo Fisher Scientific, USA). The reaction was performed in the presence of different length primers for the simultaneous identification of both SNPs. Products of the reaction were separated by capillary electrophoresis in ABI PRISM^®^ 3100 Genetic Analyzer and analyzed by GeneMapper^®^ Software v. 4.0 (Thermo Fisher Scientific, USA). The genotyping procedure of FTO SNPs has previously been described in detail at Kolačkov et al. [14].

### Statistical analysis

Deviation of genotype distribution from Hardy–Weinberg equilibrium was analyzed using the Chi square test. Shapiro–Wilk’s test was used to test the assumption of normality. When a distribution was normal, Student’s *t* test was taken for the assessment of the statistically significant differences. Mann–Whitney’s *U* test was applied to test differences for other parameters. A p value less than 0.05 was used as a level of statistical significance. Statistical analysis was performed using the STATISTICA, ver. 13.0 medical package.

## Results

Due to small sample group of acromegalic patients and no statistical differences between analyzed parameters in relation to sex (p > 0.05), the results of female and male patients are presented together (Tables 1, 2, 3, 4).

**Table 2:** Analysis of Hardy–Weinberg equilibrium

SNP	Genotype	Observed	Expected	Hardy–Weinberg test
rs9930609	T/T	17	14.3	χ^2^ = 2.3130 p = 0.128295
	A/T	20	25.4	
	A/A	14	11.3	
rs9930506	A/A	13	11.3	χ^2^ = 0.91930 p = 0.337658
	A/G	22	25.4	
	G/G	16	14.3	

**Table 3:** Clinical parameters of the study population relative to genotypes of *FTO* rs9939609

Parameter (mean)	Genotype			*p* value		
	TT	AT	AA	TT vs. AT	AT vs. AA	TT vs. AA
BMI (kg/m^2^)	27.4	28.0	29.4	0.4645	0.3913	0.1588
Glucose (mg/dl)	102.3	92.0	98.6	0.3146	0.7264	0.6624
Total cholesterol (mg/dl)	200.4	201.7	217.0	0.5422	0.3020	0.3210
Triglyceride (mg/dl)	105.4	109.3	145.6	0.6808	0.1464	0.0807
HDL-C (mg/dl)	61.4	52.8	49.2	0.0609	0.1616	**0.0024**
LDL-C (mg/dl)	118.0	127.1	138.6	0.2931	0.6366	0.1366

*BMI* body mass index, *HDL-C* high-density lipoprotein-cholesterol, *LDL-C* low-density lipoprotein-cholesterol

**Table 4:** Clinical parameters of the study population relative to genotypes of *FTO* rs9930506

Parameter (mean)	Genotype			*p* value		
	AA	AG	GG	AA vs. AG	AG vs. GG	AA vs. GG
BMI (kg/m^2^)	28.1	27.6	29.5	0.8244	0.1433	0.3028
Glucose (mg/dl)	106.6	92.2	96.6	0.1331	0.9764	0.2278
Total cholesterol (mg/dl)	205.4	201.4	211.1	0.7848	0.5543	0.7093
Triglyceride (mg/dl)	109.2	106.7	140.5	0.6327	0.1171	0.2542
HDL-C (mg/dl)	61.2	55.5	48.3	0.3058	**0.0266**	**0.0070**
LDL-C (mg/dl)	122.3	124.7	134.8	0.6820	0.5743	0.4428

*BMI* body mass index, *HDL-C* high-density lipoprotein-cholesterol, *LDL-C* low-density lipoprotein-cholesterol

The distribution of the *FTO* rs9939609 and rs9930506 genotypes did not deviate from Hardy–Weinberg equilibrium (Table 2). The risk allele frequencies of rs9939609 (A) and rs9930506 (G) were 0.471 and 0.529, respectively.

There was no association of *FTO* gene polymorphisms with BMI and mean concentrations of glucose, total cholesterol, LDL cholesterol and triglyceride. The statistical analysis revealed that the risk alleles were associated with decreased HDL concentration.

**Fig. 1:**
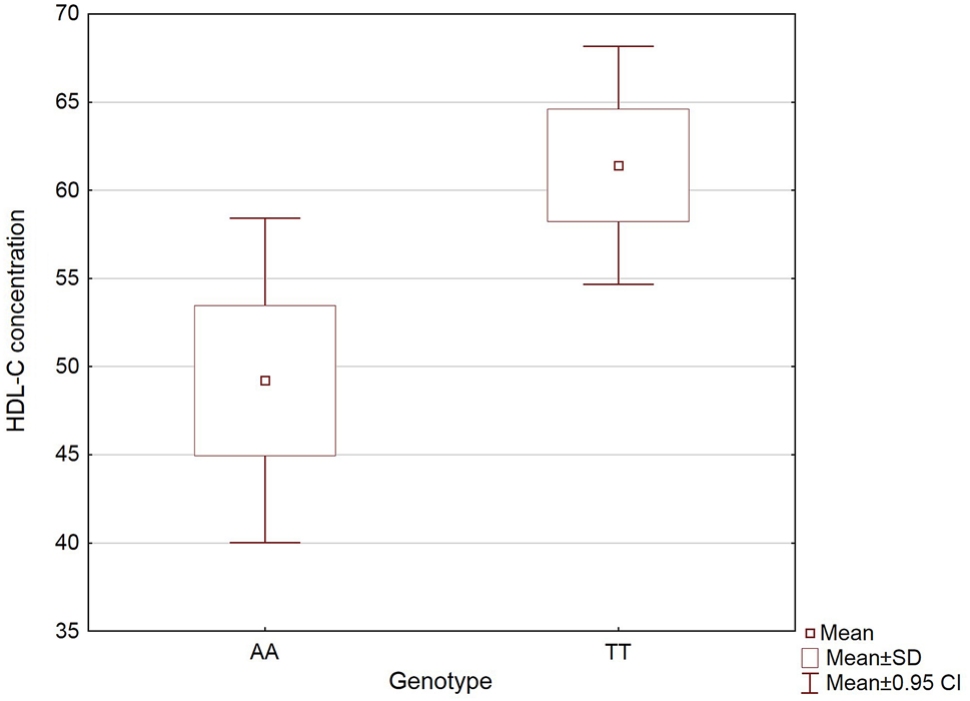
Mean HDL-C concentrations of acromegaly patients with AA and TT genotypes of *FTO* rs9939609

It has been reported that homozygotes for the rs9939609 risk allele A had 1.25-fold lower HDL-C concentration than carriers of the TT genotype. A statistically significant difference was found between AA and TT genotypes (p = 0.0024) and a trend towards significance between TT and AT genotypes (p = 0.0609) (Fig. 1). All data representing summary statistics related to *FTO* rs9939609 and clinical parameters are presented in Table 3.

**Fig. 2:**
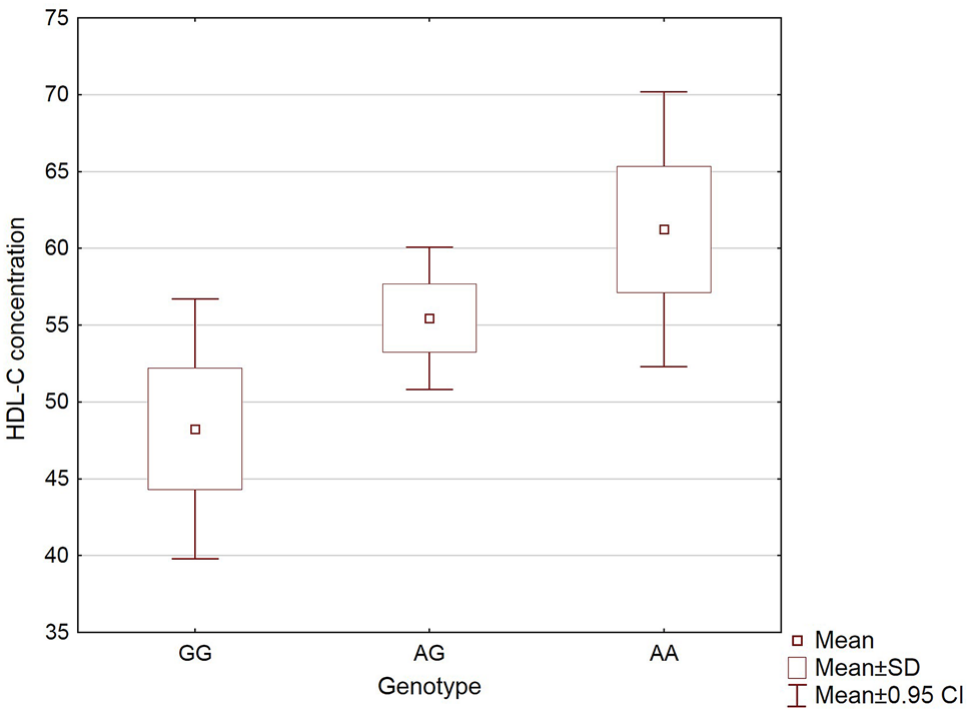
Mean HDL-C concentrations of acromegaly patients with GG, AG and AA genotypes of *FTO* rs9930506

The estimated average decrease in HDL-C concentration per risk allele for rs9930506 was 11.2%. However, statistically significant differences were observed only between AG and GG as well as AA and GG genotypes (Fig. 2). Homozygotes for the rs9930506 risk allele had 1.27-fold lower HDL-C concentration than AA carriers. All data representing summary statistics related to *FTO* rs9930506 and clinical parameters are presented in Table 4.

## Discussion

Acromegaly is a rare endocrine disease which delayed diagnosis or inappropriate treatment results in the development of severe complications. This disease is characterized by two-fold higher mortality than in the general population. The leading causes of morbidity and mortality in patients with acromegaly are cardiovascular diseases [3].

Many population-based studies suggest a role of genetic ground in CVD and associated metabolic disturbances [15]. According to a few studies, the polymorphisms of *FTO* gene are associated with obesity, as well as with an increased risk of CVD [7–9]. Although, there is no data about such contribution among acromegalic patients, some research indicates an impact of other polymorphisms. Turgut at al. showed a significant association between Leptin Receptor Gene polymorphism and carotid Intima Media Thickness (cIMT) which may result in higher risk of development of early atherosclerosis in acromegaly [16], and in another study, that Growth Hormone Receptor gene polymorphisms may be a risk factor of higher plasma insulin concentration, higher BMI and increased systolic blood pressure [17]. Oguz at al. indicated the relationship between intercellular adhesion molecule (ICAM) gene polymorphism and hypertension, higher fasting plasma glucose and HDL-C [18]. Furthermore, it is suggested that IGF-1, which is the main mediator of growth hormone actions, may also mediate actions of *FTO* gene. Biological evidence is based on the abundant expression of *FTO* in the regions of brain, which are also key organs for the GH-IGF-1 axis. Moreover, the action of *FTO* gene as well as GH is the most intense in youth, and gradually diminished with advanced age. The data revealed that risk allele carriers have lower IGF-1 concentrations which can lead to obesity [12]. Above mentioned reports were a reason to undertake our research.

To the best of our knowledge this study is the first one showing an impact of *FTO* gene polymorphisms on CVD-associated parameters in acromegaly.

At first, we assessed the risk allele frequencies of rs9939609 and rs9930506 polymorphisms, which were 0.471 and 0.529, respectively. In our study they were found to be higher than in HapMap CEU population (0.460 and 0.486, respectively) [19]. The observed differences in allele frequencies may be caused by specificity of the study group. There are limited data available about the MAFs (*minor allele frequency*) of the *FTO* in Polish population. In one of the available studies MAF of rs9939609 in population of Lower Silesia was 0.44 [20]. For now, there is no possibility to compare results concerning rs9930506. We have demonstrated that in group of patients with acromegaly there is no association of *FTO* gene polymorphisms with BMI and concentrations of glucose, total cholesterol, LDL-C and triglyceride. The risk alleles were associated with decreased HDL concentration and this finding is compatible with available bibliographic database [7, 8, 21]. In our study, the statistically significant differences between HDL-C concentrations among carriers of different genotypes referred to both analyzed SNPs. It was found that homozygotes for the risk allele had about 1.25-fold lower HDL-C concentration than homozygotes of another allele.

The lowered HDL-C concentration is positively correlated with CVD risk. According to epidemiological date, a 1% decrease in HDL-C is associated with a 2–3% increase in CVD risk. Anti-atherogenic action of HDL results from its anti-inflammatory and antioxidant properties. Among the causes of lower HDL-C concentration are: overweight and obesity, physical inactivity, smoking, type 2 diabetes, elevated triglyceride concentration and genetic factors. The latter one are responsible for about 50% of the variability of HDL-C concentrations. The FTO gene variants may be also a genetic background of its variability [22].

To date, there are few studies showing an association between the FTO gene polymorphisms and CVD risk. According to those available studies the presence of risk allele leads to a decreased HDL concentration. In 2008 Freathy et al. reported such association in a large group of Europeans [21]. This associations was also revealed in studies on patients with abnormal glucose metabolism. Additionally, possession of the A allele of rs9939609 contributes to higher cardiovascular morbidity and mortality than in TT homozygotes, what was revealed during the follow-up research [7, 8]. A large meta-analysis confirmed the significant association of the rs9939609 polymorphism with CVD risk, which was not mediated by changes in BMI [9]. The limitation factors of this study could be relatively small sample group and lack of matched control group.

In conclusion, out results are in accordance with previous findings concerning an influence of *FTO* gene variants on HDL-cholesterol concentration. Despite relatively small sample group, it was shown that analyzed polymorphisms are associated with variability of HDL-cholesterol concentrations which may lead to increased CVD risk in patients with acromegaly who are risk-allele carriers. Nevertheless to provide more precise evidence, further investigation on larger sample size is required.
